# Direct Percutaneous Endoscopic Jejunostomy: High Completion Rates with Selective Use of a Long Drainage Access Needle

**DOI:** 10.1155/2009/520879

**Published:** 2009-06-16

**Authors:** G. W. Moran, N. C. Fisher

**Affiliations:** Gastroenterology Department, Russells Hall Hospital, Dudley, West Midlands DY1 2HQ, UK

## Abstract

*Background*. Direct percutaneous endoscopic jejunostomy (DPEJ) insertion is a useful technique for artificial nutritional support in selected patients. However, it is technically difficult and most case series report significant procedural failure rates. *Methods*. We reviewed our case series of DPEJ insertions, done in a tertiary care referral centre from 2002 to 2008. Patients were selected for DPEJ if they required artificial enteric nutritional support but were unsuitable for endoscopic gastrostomy. Our technique includes selective usage of a long drainage access needle for gut luminal puncture, selective fluoroscopic guidance and selective usage of general anaesthesia. *Results*. Of 40 consecutive patients undergoing attempted DPEJ insertion, 39/40 (97.5%) had a successful procedure. Sixteen cases (40%) required the drainage access needle for completion, nineteen cases (47.5%) were done with fluoroscopy, and five cases (12.5%) were done under general anaesthesia. There were no procedural complications. *Conclusions*. This technique led to a high completion rate and low complication rate. With appropriate care and expertise, DPEJ insertion is reliable and safe.

## 1. Introduction

Artificial enteric nutritional support is vital in the management of subjects who are unable to maintain oral nutrition during or following conditions such as upper gastrointestinal malignancy surgical resection, cerebrovascular disease, or dysmotility [1]. In some of these situations, postpyloric feeding is required [2]. For delivery of postpyloric feeding, the direct percutaneous endoscopic jejunostomy (DPEJ) is the device of choice for many clinicians because it a secure, high calibre device (typically 15 Fr or more) which is unlikely to block and cannot migrate, in contrast to other devices that are easier to place such as nasojejunal or PEG-J tubes [3–6]. However its limitations are that it is generally more difficult to place and with a higher risk of complications, so its usage is not widespread. 

In the past six years at our institution we have adopted the DPEJ as the device of choice for artificial nutrition in a small group of highly selected patients, principally those with complications of advanced upper GI malignancy. During this time our technique has been refined to include selective usage of a long drainage access needle for enteric access, in order to maximise technical success rate and minimise risk of complications. We describe here our technique and outcome. Data for this review were obtained by case note review of all patients on a prospectively held database of attempted DPEJ insertions.

## 2. Patients and Methods

### 2.1. Patient Selection

Patients were considered for DPEJ as the primary means of artificial nutritional support if they were unable to maintain nutrition orally and if conventional endoscopic gastrostomy insertion was inappropriate (because of gastric malignancy, resection or dysmotility). All patients had a history of weight loss of >5% previous body weight. For patients with malignancy, obstructing lesions were managed with enteric stents, and DPEJs were reserved for cases not suitable for stenting. DPEJ insertion was avoided if active respiratory infection or failure was present, and in patients in the terminal phase of malignant disease, unless there was an explicit intention of using a DPEJ to allow a patient to be discharged home for terminal palliative care.

### 2.2. Technique—General Considerations

Conscious sedation is used where possible, with intravenous midazolam with or without pethidine. General anaesthesia is used in selected cases either for patient anxiety or after a failed earlier attempt under conscious sediation. Antibiotic prophylaxis with 2.2 g coamoxiclav is used. Intravenous hyoscine is used during the procedure, immediately prior to trochar insertion.

### 2.3. DPEJ Insertion

The technique is a modification of the original DPEJ procedure as described by Shike et al. [7]. The choice of endoscope used depends upon whether previous upper GI surgery has been done; if the upper GI tract is intact, an enteroscope (with overtube) is usually needed; shorter endoscopes may be used if part of the upper GI tract has been resected or anastomosed [8]. Our practice is to always have fluoroscopy available during the procedure. 

**Figure 1:**
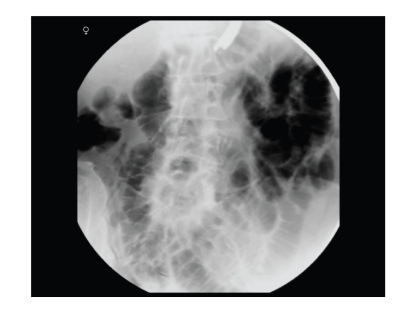
Fluoroscopic image localising endoscope position in the proximal jejunum (this patient has undergone gastrectomy).

**Figure 2:**
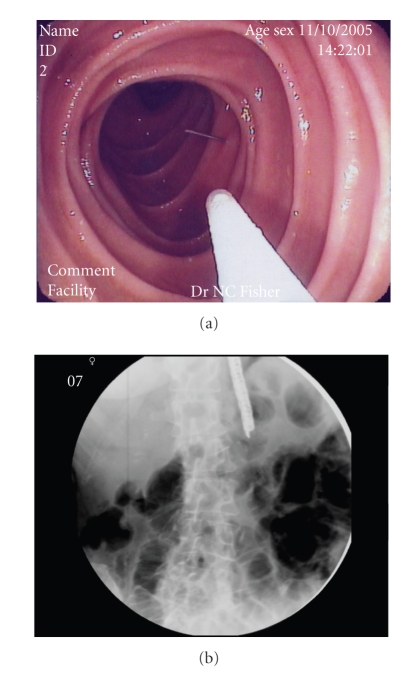
A 21G needle is advanced into the gut lumen with intermittent fluoroscopic guidance (panels (a) and (b)).

**Figure 3:**
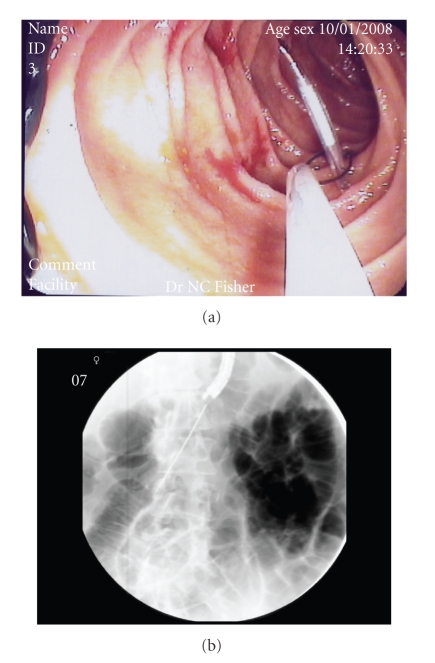
A Kellett drainage access needle is then inserted along a similar entry path to the 21G needle, prior to snaring (panels (a) and (b)).

**Figure 4:**
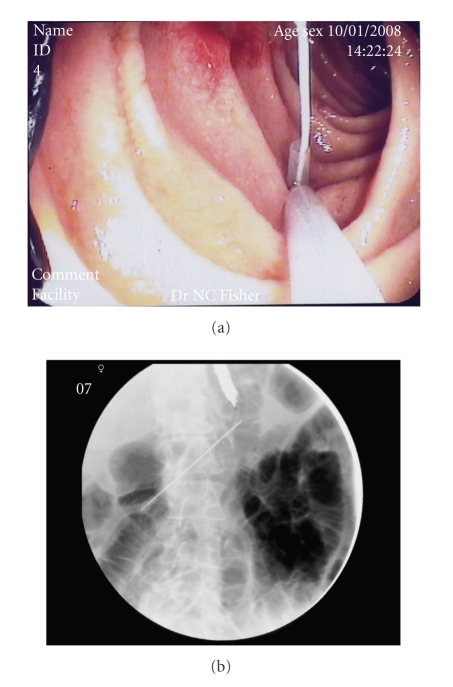
A cotton thread is passed into the Kellett needle and through the snare, following which the procedure is completed using the same technique as a conventional PEG.

The endoscope is advanced into the first loop of jejunum; with experience, this loop is usually readily identifiable. The endoscope position can usually be confirmed by finger indentation in the left upper quadrant or with fluoroscopy (if a gastrojejunostomy has been done then the endoscope position is more variable). Transillumination may also be seen here but we do not consider this a prerequisite to trochar insertion. The abdominal wall is then cleansed. Local anaesthetic is inserted at the indentation site, and a 21G pilot needle (4 cm long) is advanced and is used to search for the jejunal lumen. At this point intermittent fluoroscopy can be used to help guide the needle into the gut lumen immediately in front of the endoscope tip (Figures 1  and 2). We also use continuous aspiration on the pilot needle; when air is aspirated the needle should be endoscopically visible at the same time to confirm entry into the correct bowel loop and to help exclude puncture of interposed loops of bowel. If the needle is not visible after aspirating air then it may be in a superimposed loop of gut, and the endoscope may need to be moved into a different loop to achieve a safe puncture site. In our practice we do not generally have rotational fluoroscopy available so judgment on a safe puncture site requires interpretation of air contrast in the gut on fluoroscopy, use of air aspiration on the pilot needle and transillumination or finger indentation. After an appropriate puncture site has been identified, a trochar or drainage access needle is advanced alongside or in place of the 21G needle. If there is uncertainty regarding the distance from the abdominal wall to the gut lumen, for example, when the gut lumen cannot be entered with a 21G needle, then fluoroscopy may be helpful to ensure that there are no loops of bowel between the jejunal loop and the abdominal wall and to help guide the direction of the needle. In this situation, several needle passes with intermittent fluoroscopy may be required for successful puncture. Once the gut has been successfuly punctured, the trochar or needle is then snared, and the procedure can be completed as for a conventional PEG insertion (Figures 3  and 4). Our unit normally uses a Fresenius 15 Fr PEG kit for these procedures.

**Figure 5:**
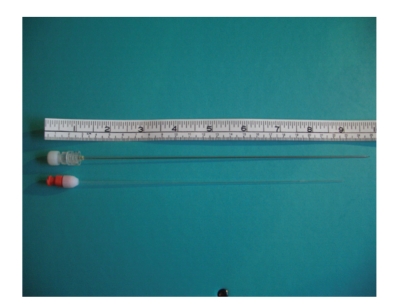
Kellett drainage access needle.

The principal variation on previous techniques that we use is the drainage access needle as an alternative to the conventional PEG trochar. We use a Kellett needle (Cook, UK; Figure 5) although other similar needles are commercially available. This is a 15 cm needle with a 19G stylet, and a 5 Fr outer sheath which was first described for use in percutaneous cholecystlithotomy [9]. Being longer than a conventional PEG trochar it is often useful because its longer “reach” may facilitate gut puncture in patients who are overweight or have omental malignancy; furthermore with its narrow gauge (in comparison with some PEG trochars), it is relatively easy to puncture a mobile loop of small bowel with this needle. Thus if the jejunum is tethered to or very close to the abdominal wall, then we use a conventional PEG trochar, but if difficulty in puncturing the gut lumen is anticipated then a drainage access needle is used. As with the “searching” 21G needle, intermittent fluoroscopy is helpful with this needle to guide its directional placement and to help ensure that no interposed loops of bowel are punctured. One important technical point is that only a single thread of cotton (or nylon) will pass through the 5 Fr sheath, which means that the loop used in most “PEG” kits to advance through the trochar sheath has to be cut at its apex, and a single thread only is passed into the jejunum prior to snaring. However, in our experience this has never prevented successful completion of the DPEJ procedure. Once the DPEJ has been placed, we do not routinely do any further imaging procedure and we generally start feeding within 24 hours.

## 3. Results

Forty patients (23 males, 17 females; median age 69) had attempted DPEJ insertion between January 2002 and April 2008. Two patients prior to this series had unsuccessful attempts without any further procedure and were not, therefore, included in this review. A further two patients within this case series had an unsuccessful procedure at the first attempt (due to restlessness), but then had a successful DPEJ with general anaesthesia at the second attempt. Of the remaining 38 cases in this series, all but one (see below) were successful at the first attempt. 

Of the 40 patients in this series, 27 (67%) had previous or current upper GI malignancy (oesophagus 7; stomach 17; pancreas 3). Of the 27 patients with a history of malignancy, 12 (44%) had incurable disease at presentation and did not have surgery, and 15 (56%) had undergone surgery with resection of malignancy. Of the 15 postoperative cases, 11/15 (73%) had recurrent malignancy prior to DPEJ insertion; in these cases the median interval from surgery to DPEJ insertion was 12 months. Thus 23 out of 27 cases (85%) with malignancy had inoperable or recurrent disease. The remaining 4 cases without recurrent malignancy had DPEJ insertion because of inability to maintain oral nutrition despite curative surgery. 

The 13 cases without GI malignancy comprised 3 cases of gastroparesis (one diabetic, two idiopathic); 3 cases of acute cerebrovascular disease (all had previous gastrectomy for peptic ulcer disease); 3 postoperative cases with surgical complications of benign disease (2 perforated duodenal ulcer with postoperative fistula or gastroparesis and 1 duodenal stenosis after aneurysm repair); 2 cases of cerebral palsy with hiatus hernia and reflux disease; 2 cases of complicated pancreatitis. Indications for DPEJ are summarised in Table 1.

DPEJ insertion was done with a drainage access needle in 16/40 cases (40%). Fluoroscopy was used in 19/40 cases (47%), with a median fluoroscopy time of 1.2 minutes. General anaesthesia was used in 5/40 cases (12%). Remaining cases were done using a conventional 10 cm PEG trochar, without fluoroscopy and with conscious sedation. The median endoscopic procedure time was 20 minutes (range 15–33). One case of failed DPEJ insertion was in a patient with late postoperative dysmotility after oesophagogastrectomy; in this patient the jejunum could not be accessed safely, despite fluoroscopy because of interposed distended colon, and no further was attempted thereafter. 

There were no consistent clinical differences between cases that did or did not require the drainage access needle for success. In 21 patients who had had previous upper GI resection or anastomosis for any reason, 8/21 (35%) had their DPEJ done with this needle, which was similar to 8/18 (44%) done with the drainage access needle in the remaining 18 patients who had had no resection. However, certain clinical situations made the procedure more likely to succeed with the drainage access needle; three examples are given in Table 2. 

There were no technical complications encountered, that is, no cases of perforation, peritonitis, or gastrointestinal haemorrhage, and no patients required early DPEJ removal for peristomal sepsis or other complication. However, overall 30 day mortality was relatively high at 14/40 (35%), reflected advanced malignancy in most cases. The median survival in patients with incurable malignancy, including those with postoperative recurrent disease (23 patients in total), was 30 days (range 9 to 83). In patients with benign indications for DPEJ insertion (16 patients in total), the 30-day mortality was 3/16 (19%); each of these early deaths occurred in patients with acute cerebrovascular disease and prior gastrectomy (two had aspiration pneumonia and one had a new cerebrovascular event). Of the remaining 13 patients with benign indications for DPEJ, 12 are still alive at the time of this review (median follow-up 36 months). 

Most patients (30/39, 77%) were discharged from hospital after DPEJ insertion and most (27/30, 90%) continued to use the DPEJ for nutritional support following discharge and until death; in the remaining 2 cases it was no longer needed following discharge. 

DPEJs were removed in 8 cases; all of these patients had benign disease or curative surgery. In 3 cases the DPEJ was removed at the time of further surgery for benign disease in 3 cases the DPEJ was no longer required; in 2 cases there was seepage of bilious contents from the DPEJ site in patients where requirement for continued support was equivocal (in these cases the DPEJ was removed at 5 and 6 months resp.). The DPEJs were removed by traction following endoscopic snaring, except in one case, where a “cut and push” technique was used, without further complication.

## 4. Discussion

DPEJ remains a technique in evolution, with varying techniques described by different authors in recent years. Variations include the types of endoscope and PEG kits used, usage (or otherwise) of fluoroscopy and/ or transillumination, and type of needle used for entry into the gut lumen. In our case series we aimed to make best use of all available alternatives in order to maximise success rate, and this resulted in a high overall success rate compared with previously published series, where technical success rates ranged from 68% to 95% [5, 9–12]. The procedural variations which we believe to be most important in achieving a high success rate in this series were firstly the selective usage of the drainage access needle for gut puncture, secondly the routine availability of fluoroscopy, and thirdly the selective usage of general anaesthesia. 

Most previously described techniques for DPEJ insertion do not specify length or needle gauge of trochar used, although 16G and 18G needles have been described [11, 13]. We are unaware of any other reports of usage of the Kellett or similar length needles. Conventional enteric access trochars for gastrostomy or jejunostomy tend to be shorter (8 cm or less) and higher gauge (14G–18G) which may be difficult to puncture a mobile or deep loop of gut with. Techniques that may help gut puncture with conventional trochars include using the endoscope to mobilise the jejunum towards the abdominal wall (made easier with a double balloon enteroscope) and snaring the pilot 21G needle to help “fix” the gut wall against the abdominal wall and on occasions we have used these techniques [9, 14]. However, situations such as omental malignancy, hiatus hernia, or obesity remain a challenge and having adopted the drainage access needle early in our practice we have found this to be invaluable in such situations. 

We found fluoroscopy to be particularly helpful where the drainage access needle was used and believe that this improves the safety of the procedure. Other published series of percutaneous jejunostomy insertion with fluoroscopy do not report bowel perforation, in contrast to some series without fluoroscopy, where occasional perforations are described [13, 15, 16]. 

Some clinicians recommend transillumination to guide abdominal wall puncture and give failure to transilluminate as a reason for procedural failure [5, 9, 12, 17]. In our practice we do not routinely use transillumination, although we recommend fluoroscopy in cases where neither transillumination nor close finger indentation can be seen. 

As with other DPEJ series, the presence of surgical scars following previous bowel resection did not pose a significant hazard in our experience; indeed since the small bowel is likely to be tethered to the abdominal wall in such cases, DPEJ insertion may actually be easier [11, 18]. 

Regarding postprocedure complications, the possibility of aspiration pneumonia is a particular concern. Avoidance of this complication requires careful case selection and a technique that minimises procedure time. The only two patients in our series who died of aspiration pneumonia both had benign disease and prior gastrectomy. Others have noted a higher incidence of aspiration pneumonia in patients undergoing DPEJ after gastrectomy [18]. Delayed complications in the form of peristomal leakage occurred in two patients in our series. This has been noted by others and is a potential concern in patients with benign disease, where long-term feeding is required [9]. However, should DPEJ removal be required, then our usage of 15 Fr PEG catheters has not so far led to any cases of persisting enterocutaneous fistula or other complications, unlike some other series where larger diameter catheters had been used [17, 19]. 

Our 30-day mortality was high at 35% but this reflected advanced malignancy in most cases, and there were no cases of death resulting from technical complications. There is little data on 30-day mortality in other similar case series in the literature. In two case series of similar size, but each with fewer cases of advanced malignancy, mortality rates of 17% and 29%, respectively, were reported [11, 16]. In two larger case series, 30-day mortality rates are not reported [5, 12]. The cost effectiveness of artificial nutrition via DPEJ feeding in patients with advanced malignancy and limited life expectancy remains uncertain and deserving of further study, but we believe that the technique can be an important aid to palliation in appropriately selected cases.

In summary, where postpyloric feeding is required for artificial nutritional support, the DPEJ technique that we have described combines a high procedural success rate with a low complication rate. Selective usage of a long drainage access needle can help maximise the success rates and thus clinical applications of DPEJ insertion.

## Figures and Tables

**Table 1:** Indications for DPEJ insertion in our cohort of patients.

Case description	Number
Oesophageal malignancy, incurable	1
Gastric malignancy, incurable	10
Pancreatic malignancy, incurable	3
Oesophageal malignancy, postoperative recurrence	5
Gastric malignancy, postoperative recurrence	4
Upper GI malignancy, postoperative malnutrition	4
Postoperative malnutrition (benign disease)	3
Acute cerebrovascular disease (stomach resected)	3
Gastric dysmotility	3
Cerebral palsy	2
Pancreatitis	2

**Table 2:** Case summaries of three examples of DPEJ insertion where particular procedural difficulties were encountered, and where the drainage access needle was helpful in ensuring a successful outcome. No case developed any complication.

Case	Procedural challenge	Technique
Male, 25. Severe cerebral palsy with dysphagia and recurrent aspiration pneumonia	Large paraoesophageal hernia with proximal jejunum in thorax	Enteroscope with overtube used. Jejunum punctured within thorax (via diaphragmatic hiatus from abdominal wall approach) with fluoroscopic guidance
Female, 53. Pancreatic cancer with partial duodenal obstruction and peritoneal metastases	Large volume of omental malignancy and moderate amount of ascites	Enteroscope used. Deep loop of jejunum punctured with fluoroscopic guidance
Female, 65. Gastric linitis plastica with peritoneal metastases	Overweight patient with mobile jejunum	Enteroscope used. Jejunum punctured with fluoroscopic guidance
